# Is the author recognition test a useful metric for native and non-native English speakers? An item response theory analysis

**DOI:** 10.3758/s13428-021-01556-y

**Published:** 2021-04-05

**Authors:** Sean Patrick McCarron, Victor Kuperman

**Affiliations:** 1 Department of Linguistics and Languages, McMaster University, Hamilton, ON Canada; 2 Department of Experimental Psychology, University of Oxford, Anna Watts Building, Woodstock Rd, Oxford, OX2 6GG UK

**Keywords:** Author recognition test, Item response theory, Print exposure, Literacy, Reading proficiency

## Abstract

Studies of reading have shown the “Matthew effect” of exposure to print on reading skill: poor readers avoid reading, and ability develops more slowly compared to peers, while good readers improve more quickly through increased exposure. Yet it is difficult to determine just how much an individual reads. The Author Recognition Test (ART, Stanovich & West *Reading Research Quarterly, 24*(4), 402-433, 1989) and its multilingual adaptations are often used for quantifying exposure to print and have shown high validity and reliability in proficient readers in their dominant language (L1). When studying bilingualism and second language acquisition, it is ideal to have a single test which is equally reliable for all cohorts for comparison, but it is unclear whether ART is effective for speakers of English as a foreign language (L2). This study assesses the reliability of ART in English-medium university and college students with different language backgrounds. Following Moore and Gordon (*Behavior Research Methods*, *47*(4), 1095-1109, 2015), we use item response theory (IRT) to determine how informative the test and its items are. Results showed an expected gradient in ART performance, with L1 speakers showing higher scores than L2 speakers of English, university students showing higher scores than college students, and both cohorts performing better than students in an English as a second language (ESL) university pre-admission program. IRT analyses further revealed that ART is not an informative measure for L2 speakers of English, as most L2 participants show a floor effect. Reasons for this unreliability are discussed, as are alternative measures of print exposure.

## Introduction

It is a long-standing observation that reading proficiency stands in a reciprocal causal relation to the amount of reading one undertakes in their free time (McQuillan & Au, 2001; Mol & Bus, 2011; Paulson, 2006). Naturally, how much voluntary reading a person does is at least partially influenced by their attitude toward reading as a pastime (Kush, Watkins, & Brookhart, 2005). At the earliest stages of reading development, the ability to decode new words using phonological knowledge is a particularly important skill which sets apart higher- and lower-skilled readers (Perfetti, Beck, Bell, & Hughes, 1987; Tunmer & Nesdale, 1985). This ability gap leads to a “rich-get-richer and poor-get-poorer” phenomenon, whereby lower-skilled readers, discouraged or unable to derive enjoyment from reading, are slow to develop reading skills and expanded vocabulary knowledge, whereas skilled readers avidly consume literary material and thus reap the benefits. This bidirectional relationship is often referred to as a “Matthew effect” for reading (Kempe, Eriksson-Gustavsson, & Samuelsson, 2011; Stanovich, 1986). For a discussion on possible mediating factors see also Bast and Reitsma (1998), Pfost, Hattie, Dörfler, and Artelt (2014), and for additional discussion on the issues surrounding psychometric analyses of a possible Matthew effect for reading see also Protopapas, Parrila, and Simos (2016) and Protopapas, Sideridis, Mouzaki, and Simos (2011). Understanding this relationship makes the operationalization and measurement of the amount of reading by an individual (their “exposure to print”) an important goal for the study of reading.

One possibility for assessing an individual’s exposure to print is to administer questionnaires which collect subjective judgments from respondents on the amount, genre diversity, or complexity of reading that they do, as well as an evaluation of their own reading proficiency. An example of this is the Reading Habits Questionnaire developed by Acheson et al. (2008). In this self-evaluation, participants are asked to report how much time they spend reading and writing in an average week, as well as whether they think they read more or less than their peers. Similar questionnaires have been administered for developmental college students (Sheorey & Mokhtari, 1994), young readers in England (Cain & Oakhill, 2011), teaching candidates (Benevides & Peterson, 2010), and a comparison between White and Asian Americans (Scales & Rhee, 2001). Self-reported evaluations of this kind, however, run the risk of interference from social desirability factors, which may encourage respondents to overstate the breadth of their reading habits.

A complementary method of establishing how much an individual reads is through proxy tests designed to determine their level of print exposure. Perhaps the best-known test of exposure to print is the Author Recognition Test (ART), first developed by Stanovich and West (1989). This test presents participants with a list of author names and distractor (“foil”) names, but which could nevertheless plausibly be believed to be names of real-world authors. Participants are asked to identify only which names belong to real authors by indicating with a checkmark, and to ignore any names which are not believed to be those of published authors. The resulting score is calculated by subtracting the number of foils incorrectly selected from the number of correct responses. Similarly, a magazine recognition test (MRT) has also been used for measuring one’s knowledge of magazine titles (Stanovich & West, 1989) as a means of assessing print exposure through more popular media. Another related measure called the Title Recognition Test (TRT) assesses knowledge of book titles (Cunningham & Stanovich, 1990). This method has been used to compare disabled versus non-disabled grade school readers (McBride-Chang, Manis, Seidenberg, & Custodio, 1993).

The linking hypothesis of such tests is that the quantity of reading materials (e.g., books or magazines) that one is exposed to correlates with one’s reading skill (Mol & Bus, 2011; Weinberger, 1996). Importantly, the tests do not assume that respondents have read the specific authors, magazines, or books about which they are queried. Instead, the assumption is that a greater amount of reading leads to a greater awareness of the existing literature and reading sources, which translates into higher recognition scores. This greater awareness and its ancillary benefits have been referred to as “cultural capital” (Bourdieu, 1986; Tunmer & Chapman, 2006), discussed in more detail below.

A large body of research has confirmed the ART’s usefulness as a predictor of proficiency in lexical tasks: as will become important below, most of this work involved university-level native readers of English. For example, scores on the ART have been shown to correlate positively with vocabulary size (e.g., Krashen & Kim, 1998; Lee, Krashen, & Tse, 1997; Martin-Chang & Gould, 2008; Rodrigo, McQuillan, & Krashen, 1996; West & Stanovich, 1991), speed or accuracy of reading words, sentences, or passages (e.g., Acheson, Wells, & MacDonald, 2008; Choi, Lowder, Ferreira & Henderson, 2015; Kuperman, Matsuki, & Van Dyke, 2018; Martin-Chang & Gould, 2008; Moore & Gordon, 2015), and reading comprehension (Cipielewski & Stanovich, 1992; Landi, 2010). Furthermore, Unsworth and Pexman (2003) found that those who scored higher on the ART did not show regularity effects in lexical decision and phonological lexical decision tasks, suggesting they had better mental access to phonological information when reading. The ART is also popular because it can be administered in only a matter of minutes, making it one of the fastest ways to ascertain an individual's approximate level of reading proficiency.

Given the popularity and demonstrated validity of the ART for proficient native readers of English, much work has been conducted to establish its reliability in this population. For example, Acheson, Wells, and MacDonald (2008) used the student population of the University of Wisconsin to determine that the original ART developed 20 years earlier contained many names of authors who were no longer well known to college students. In response, they developed a new version of the ART which consisted of 130 items (65 author names and 65 foils) and kept only 15 of the original author names. A more recent psychometric study of 1012 students at the University of North Carolina by Moore and Gordon (2015) used item response theory (IRT) to evaluate the discriminative value of each item on the ART, i.e. how much a correct or incorrect response to each author name and each foil distinguishes readers with different levels of exposure to print (see detailed discussion below). The outcome of this study was the determination of a discriminative value for each item and a proposed reduction of the ART item list from 130 (65 author names and 65 foils) to 100 of the most discriminative items (50 author names and 50 foils). Moore and Gordon (2015) evaluated the reliability of the abridged version of ART and confirmed its validity as a predictor of eye movements registered during reading for comprehension (N=789, all correlations between mean gaze duration and the 100-item ART score highly significant at *p* < .001), see also Choi et al. (2015) and Kuperman and McCarron (2019).

Furthermore, researchers have created ART versions for Hebrew (Shatil, Share, & Levin, 2000), Dutch (Vander Beken & Brysbaert, 2018), Korean (Kim & Krashen, 1998a; Lee et al., 2019), and Chinese (Chen & Fang, 2015), as well as those intended specifically for readers in the United Kingdom (Masterson & Hayes, 2007) and Canada (Chateau & Jared, 2000; Sénéchal et al., 1996). Additionally, ARTs for English-speaking children (sometimes called a Children’s Author Recognition Test or CART) have been developed and implemented (Cipielewski & Stanovich, 1992; Ricketts, Nation, & Bishop, 2007; Stainthorp, 1997). Self-administered versions of the ART have even been created which contain no foils and were shown to still be a strong predictor of vocabulary size despite participants being informed that all names listed were of published authors (Krashen & Kim, 1998).

It can be confidently stated that the ART is a reliable and valid method for examining native speakers of English at the university level, and that it is adaptable to other languages and other populations. What is less certain, however, is whether or not the ART can be used *without adaptation* as a reliable tool in studies that involve comparisons between native and non-native readers of English, or between individuals widely different in their English reading proficiency—from university students to college students^1^ to students enrolled in ESL classes. Such comparisons are essential for answering a number of critical theoretical and practical questions in psychology and education. Thus, these fields need a tool that can be used as a uniform instrument for comparing exposure to print across populations with variability in reading skills in their L1 and L2. The efficacy of the ART in evaluating these additional populations compared to native English university students will be the primary question of interest for the present paper.

There are indeed examples of studies which have used the ART and similar methods of evaluating print exposure in native English-speaking cohorts with different proficiency levels. These include unskilled versus skilled child readers (Ricketts, Nation, & Bishop, 2007), older versus college-age readers (Stanovich, West, & Harrison, 1995), and high- versus low-skilled postsecondary readers (Lewellen, Goldinger, Pisoni, & Greene, 1993). Moreover, the English version of the ART has also been used to predict literacy skill in second-language learners of English (Kim & Krashen, 1998b; McQuillan, 2006; Miller Guron & Lundberg, 2003; Stuart, 2004). Importantly, this comparative research effort may be jeopardized if the tool used for assessment is unreliable for use in at least some populations under comparison. To our knowledge, no systematic psychometric analysis has been conducted to test the ART’s reliability in native English speakers with a below-university level of reading proficiency, or in non-native readers of English. Our study aims to fill this gap.

It is sensible to expect that the ART’s reliability will vary across populations. One reason is that the ART item selection is most representative of fiction authors writing in English (e.g., James Joyce, Ernest Hemingway) and more generally authors belonging to the Western literary tradition, if not necessarily those writing in English (e.g., Umberto Eco). In the case of non-native speakers of English, it is a distinct possibility that the materials they predominantly read are either not in English or—if they are in English—are not represented in the item selection of the ART. Second, individuals' reported time spent reading academic textbooks and fiction is known to be negatively correlated (Acheson, Wells, & MacDonald, 2008): for non-native speakers enrolled in an English-medium educational program, their assigned reading may be largely academic and their exposure to fiction may be comparatively limited. This generally results in lower performance on a test like ART due to the specificity of the English reading material to which L2 readers are exposed. In this case, differences in the ART scores may emerge because the English-language ART may not equally tap into the cultural capital of diverse populations.

The goal of this paper is to assess the reliability of the ART in five samples: (i) native English university-level readers; (ii) native English college-level readers; (iii)–(iv) non-native university- and college-level readers; and (v) non-native English readers enrolled in an ESL year-long pre-admission program at a university. This coverage enables us to assess how the ART’s reliability is influenced by both the language background and variability in educational level, roughly equivalent to variability in reading proficiency. We deliberately concentrate on the group-level analysis, where statistical estimates are made for each cohort (i)–(v) rather than an individual. We do recognize, of course, that the English proficiency and exposure to print vary within cohorts as well. Equally, our labels (L1 vs L2) do not intend to mark L1 speakers of English as pure monolinguals (few Canadians are, because of the mandatory school instruction in French) and L2 speakers as individuals who only speak their dominant language and English. Yet we do not pursue a goal of characterizing individual variability in exposure to print or drawing clear-cut comparisons between clear-cut cases of mono- and bilingualism. This is because, in practice, the determination of whether to use ART is not made at the level of an individual or a carefully controlled group, but rather at the level of a population available for testing. Our focus is the reliability of ART in large samples of Canadian students who are representative of the language backgrounds and the range of English proficiency naturally found in colleges and universities. These are the types of students that populate convenience pools at universities and provide most of the empirical base of language research.

## Methods

### Participants

A total of 1393 students participated in the study between September 2017 and April 2020: 891 university students were recruited from a convenience pool of McMaster University and 502 college students from a similar pool of Mohawk College of Applied Arts and Technology (both institutions located in Hamilton, Ontario, Canada). Participants represented five cohorts defined by varying levels of English proficiency and education, defined as (i)–(v) above. The minimum English proficiency for a non-English-speaking individual seeking admittance to McMaster University is TOEFL [Test of English as a Foreign Language] iBT [Internet-based test] score of 86 (with a minimum score of 20 in each of the four components: Reading, Writing, Speaking, and Listening) or an IELTS [International English Language Testing System] score of 6.5 (with a minimum score of 6 in all four components). These scores constrain our university cohort to 50% of all participants who completed TOEFL. Residence in an English-speaking country or English-medium academic experience in a secondary education setting for at least four years granted an exemption from this requirement. Respective cutoffs for L2 students admitted to Mohawk College are similar: 83 TOEFL iBT or 6.5 IELTS (with a minimum score of 6 in all four components). The University ESL cohort is defined by the lower threshold TOEFL score of 70 and the upper threshold of 85 (scores above that grant direct admittance without the bridging ESL program).

As part of the collection of demographic data, we asked participants to provide information about their language experience, including their first language, country of birth, and number of years spent in Canada. Tables 1 and 2 report descriptive statistics of the age at which cohorts learned English and the age at which cohorts came to Canada.

**Table 1:** Average ages at which cohorts learned English

Level of education	English	Minimum	Q1	Median	Q3	Maximum	Mean	Range
College	L1	0	0	0	0	9	0.19	9
	L2	0	5	7	11	39	8.63	39
University	ESL	0	6	8	10	18	8.07	18
	L1	0	0	0	0	6	0.02	6
	L2	0	0	6	9	22	6.36	22

**Table 2:** Average ages at which cohorts arrived in Canada

Level of education	English	Minimum	Q1	Median	Q3	Maximum	Mean	Range
College	L1	0.00	0	0	0	46	1.59	46
	L2	0.00	17	20	23	48	19.31	48
University	ESL	0.00	16	16	18	50	16.90	50
	L1	0.00	0	0	0	17	0.40	17
	L2	0.00	4	15	17	41	11.44	41

As expected, the L1 cohorts in both the university and college were predominantly born in Canada and exposed to English from birth. The data in Tables 1 and 2 demonstrate that most university and college L2 and ESL speakers started learning English early, i.e., by or in elementary school (median age 6–8 years.; third quartile 9–10 years.). The median age of arrival in Canada was around the beginning of high school for university L2 students (median age 15 years) and towards the end of after high school for ESL and college L2 students (median age 16–20 years). Proportions of self-identified L2 university and college students who arrived in Canada before the age of formal instruction (6 years) were below 30%. Thus, a typical profile of an L2 (non-ESL) speaker that emerges from these data is that of a person with an early exposure to English and, mostly, an arrival in Canada in adolescence, i.e., at an age which proponents of the critical period of second language acquisition would place *after* this period. This profile also comes with a relatively high proficiency in all faculties of the English language, as all participants met language requirements of respective institutions (see above). The ESL cohort was exposed to English relatively early, arrived in late adolescence or young adulthood, and spent only 1–2 years in Canada prior to testing.

Table 3 additionally reports the distribution of self-reported first languages spoken by participants in the university- and college-based cohorts.

**Table 3:** Most frequently spoken first languages in university and college samples

College			University	
Number	Language	Frequency	Language	Frequency
1	English	317	English	280
2	Punjabi	90	Chinese	163
3	Gujarati	64	Urdu	16
4	Chinese	29	Arabic	11
5	Hindi	23	Korean	9
6	Vietnamese	21	Russian	8
7	Arabic	20	French	7
8	Spanish	18	Punjabi	7
9	Malayalam	16	Farsi	5
10	Portuguese	15	Polish	5

Mohawk College students were compensated by participation in a lottery that randomly distributed twenty $50 gift cards for the college’s bookstore, and McMaster students were given a partial course credit. The study received ethics clearance from the McMaster Research Ethics Board (REB) (2018-033) and Mohawk REB (18-003).

### Materials

All university samples (L1, L2, and ESL) completed the 130-item version of the ART published in Acheson et al. (2008), whereas the college samples (L1 and L2) were administered the 100-item version of the ART from Moore and Gordon (2015). To make the outcomes comparable, we later rescored the 130-item ART to match the outcomes of the 100-item test version as follows. First, we only considered those 50 fiction authors (out of 65) that were included in Moore and Gordon’s abridged ART due to their informativity. Second, the foils were different in the 130-item and the 100-item ARTs. We randomly selected 50 foils (out of 65 in the 130-item ART) and only considered participants’ responses to those foils. The scores obtained by each university-based sample in the full 130-item ART and its 100-item subset defined above correlated at *r* > 0.9 (all *p*s < 0.001). We conclude that our reduction of the 130 items to 100 is highly representative of exposure to print in all university-based samples.

### Procedure

Participants began by providing informed consent and then responded to a demographic questionnaire, including information about their age, education, and first language, as well as their subjective estimate of reading, writing, listening, and speaking proficiency in English. In all cohorts, ART was part of a bigger battery, which we do not report here. The entire experimental session did not exceed 15 minutes in the college samples and 1 hour in the university samples.

For the ART component of the battery of tests, participants were presented with a checklist of names and were asked to check off only those names which they were certain belonged to a published author. Instructions to participants were the same as in Acheson et al. (2008). An individual ART score was calculated as follows: every correct indication of an author increased the score by 1 point; every incorrect indication decreased it by 1; no penalty was incurred for not indicating an existing author. At-chance performance yields a score of 0; negative scores are possible as well.

All analyses were performed in R 3.6.1 statistical software (R Core Team, 2019), and the IRT analysis was performed using the package ltm (Rizopoulos, 2006).

## Results

Table 4 summarizes the distribution of the ART score per cohort. Results show that native speakers in college performed more poorly in the ART task than those in university, and ESL students performed more poorly than non-native cohorts in both university and college (all *p*s < 0.05 in two-sample *t* tests after Bonferroni correction for multiple comparisons). All cohorts showed ART scores that were reliably different from the chance level of 0 (all *p*s < 0.05 in one-sample directional *t* tests). We also note that the ART score registered in our cohort of L1 university students is significantly weaker than that reported by Moore and Gordon (2015) in a cohort of 1102 students of the University of North Carolina at Chapel Hill (9.95 vs 13.57, *t* = −8.09, *p* < 0.001). Thus, regional differences between Canada and the USA in the ART performance are possible as well.

**Table 4:** Descriptive statistics of performance of all cohorts on ART, including cohort size *N*

Level of education	English	*N*	Mean	SD	Minimum	Maximum	Range
College	L1	283	7.63	9.76	−8	47	55
	L2	219	2.92	7.73	−9	47	56
University	ESL	183	1.45	6.26	−8	40	48
	L1	466	9.95	8.48	−10	45	55
	L2	242	5.12	8.73	−11	46	57

Internal consistency of responses within each cohort was very high (Cronbach’s alpha ranged from 0.947 to 0.965; ICC 2k for college L1 was 0.95 [CI 95% 0.95–0.96]; for college L2 it was 0.95 [CI 95% 0.95–0.96]; university ESL 0.94 [CI 95% 0.93–0.95]; university L1 0.91 [CI 95% 0.90–0.92], and for university L2 it was 0.96 [CI 95% 0.95–0.96]). We also conducted a test for unidimensionality as one of the requirements imposed by the item response theory used below. Unidimensionality of the data was indeed indicated by a confirmatory factor analysis model implemented in the library lavaan (Rosseel, 2012), which showed a good fit (the root mean square error of approximation [RMSEA] 0.055, CI 90% [0.053–0.058], *p* < 0.05 was below the cutoff recommended by Hu & Bentler, 1999).

ART scores also demonstrated external validity, as they correlated positively with reading comprehension. Kuperman and McCarron (2019) examined a relationship between ART and reading comprehension gauged as scores on the Gray Oral Reading Test (4th edition, GORT-4; Weiderholt & Bryant, 2001), passages 5–9, in college-based L1 and L2 cohorts reported in this study. In a regression model controlling for multiple other component skills of reading and the L1 versus L2 status of participants, a higher score in ART predicted a higher reading comprehension score (*F* = 25.30, *df* = 1, *p* < 0.0001): an increase of 10 points on the ART scale came with an increase of 3 points in the reading comprehension score. In the same vein, McCarron and Kuperman (2021) report a reliable effect of ART on reading comprehension scores in GORT-4 (passages 5–12) among L1 and L2 university students examined in this paper. This effect was positive and highly significant (*F*(1,221) = 20.31, *p* < 0.001), even when other component skills of reading and L1 versus L2 status were covariates in the model. An increase of 10 points in the ART score came with an increase of 1.5 points in GORT-4 scores. Rich independent evidence also demonstrates the external validity of ART scores as predictors of eye movements during reading (see the Introduction).

The raw scores in Table 4 clearly demonstrate that average performance on the ART varies by cohort; however, it is also important to understand the degree of similarity between cohorts with respect to the authors they tend to recognize. Table 5 reports the accuracy of recognition (percent correct) for every author in each cohort (columns %), sorted in decreasing order of accuracy in the L1 university cohort.

**Table 5:** Comparison of response accuracy and estimated IRT parameters across five cohorts. Column *%* indicates how frequently each name was correctly selected as an author, *α* indicates the level of *discrimination* of individual ability each name provides, while *β* indicates the *difficulty* of correctly selecting each name

No.	Author Name	University, English L1			University, English L2			University, ESL			College, English L1			College, English L2		
		%	α	β	%	α	β	%	α	β	%	α	β	%	α	β
1	Stephen King	89.89	1.17	−2.43	58.14	1.33	−0.50	25.42	1.16	1.00	78.80	0.74	−2.15	42.47	0.88	0.33
2	F. Scott Fitzgerald	84.52	0.96	−2.21	53.49	1.83	−0.30	35.03	0.81	0.72	54.77	1.48	−0.38	27.85	1.49	0.81
3	Margaret Atwood	83.23	0.64	−2.85	49.61	1.90	−0.17	19.77	1.67	1.04	43.11	2.19	0.00	16.89	1.42	1.44
4	Harper Lee	79.78	0.94	−1.84	54.26	1.40	−0.34	25.99	1.48	0.81	45.94	1.23	−0.03	25.11	1.14	1.13
5	Ernest Hemingway	78.92	0.84	−1.93	47.67	1.45	−0.09	35.59	0.81	0.69	42.40	1.71	0.05	17.35	1.67	1.28
6	George Orwell	69.03	1.20	−0.98	52.71	1.48	−0.28	20.34	2.26	0.85	50.18	1.77	−0.21	36.53	0.91	0.65
7	J. R. R. Tolkien	69.03	0.77	−1.31	49.22	1.73	−0.16	42.94	1.18	0.16	60.78	1.23	−0.65	31.05	1.49	0.67
8	Virginia Woolf	67.96	1.04	−1.01	39.53	1.65	0.19	38.98	1.14	0.34	31.45	2.35	0.35	11.87	1.76	1.61
9	E. B. White	61.51	1.04	−0.68	35.27	1.14	0.50	33.33	1.17	0.59	34.98	1.64	0.33	21.46	1.59	1.09
10	T.S. Elliot	61.08	0.93	−0.71	41.86	1.31	0.15	27.12	1.10	0.95	40.28	2.03	0.10	30.59	1.70	0.64
11	James Patterson	58.71	1.30	−0.49	34.11	1.51	0.43	17.51	1.53	1.22	41.70	1.54	0.10	19.63	1.30	1.35
12	J. D. Salinger	57.63	1.42	−0.42	43.80	1.40	0.06	40.11	1.02	0.33	39.58	1.86	0.13	21.92	1.64	1.04
13	Maya Angelou	50.97	1.44	−0.17	33.72	1.51	0.45	15.82	2.21	1.08	19.79	2.07	0.85	15.07	1.92	1.32
14	William Faulkner	47.31	1.93	−0.03	25.97	2.22	0.61	29.38	1.71	0.59	23.32	1.92	0.73	14.61	1.56	1.52
15	Tom Clancy	47.10	0.97	0.00	28.68	1.88	0.57	19.21	2.35	0.88	49.82	1.16	−0.19	18.26	1.62	1.25
16	Danielle Steel	41.72	1.20	0.22	23.26	2.49	0.68	16.38	1.97	1.11	34.28	1.69	0.34	19.63	1.95	1.06
17	Thomas Wolfe	40.86	1.10	0.28	27.91	1.17	0.86	18.08	2.28	0.95	27.92	2.54	0.45	12.33	1.78	1.57
18	Ray Bradbury	38.92	1.54	0.28	25.58	1.35	0.89	24.29	1.95	0.75	19.08	2.61	0.77	10.96	2.55	1.43
19	Samuel Beckett	36.99	1.93	0.30	21.71	2.70	0.72	14.69	2.33	1.11	20.85	2.29	0.75	13.70	1.72	1.50
20	Kurt Vonnegut	35.48	1.67	0.39	24.81	2.18	0.67	26.55	1.54	0.76	14.84	2.57	0.98	9.59	2.54	1.53
21	John Grisham	35.27	1.64	0.40	18.60	2.47	0.90	21.47	1.32	1.12	26.86	2.40	0.50	17.81	1.91	1.17
22	James Joyce	34.41	1.53	0.45	24.42	2.17	0.69	22.60	2.34	0.74	19.08	2.21	0.84	10.96	1.94	1.60
23	Toni Morrison	32.90	1.20	0.61	26.36	1.40	0.82	24.86	1.98	0.72	16.25	3.00	0.84	15.53	1.69	1.39
24	Gabriel Garcia Marquez	31.18	1.97	0.50	23.64	1.88	0.78	20.90	1.29	1.17	9.54	5.40	1.01	13.70	1.36	1.72
25	Vladimir Nabokov	28.17	1.57	0.69	22.87	2.56	0.69	19.21	2.38	0.88	17.31	2.54	0.86	14.61	2.14	1.28
26	Isaac Asimov	27.31	1.87	0.66	20.93	2.54	0.78	18.64	1.92	1.01	16.96	3.13	0.79	14.16	2.36	1.26
27	Anne McCaffrey	27.10	1.19	0.90	20.54	1.57	1.05	25.99	1.21	0.94	17.31	2.14	0.95	13.24	2.70	1.25
28	Ayn Rand	26.45	1.67	0.74	18.99	2.14	0.95	16.95	2.92	0.90	19.79	2.66	0.73	10.05	3.04	1.41
29	Sue Grafton	25.16	1.75	0.77	16.28	2.41	1.03	18.08	2.03	1.01	14.13	2.66	1.00	10.50	2.09	1.59
30	Nora Ephron	24.30	2.23	0.71	17.83	2.13	1.01	14.69	1.85	1.25	13.07	3.12	0.99	11.42	1.67	1.70
31	Judith Krantz	23.01	1.91	0.83	13.18	1.34	1.72	20.34	1.15	1.31	9.54	3.02	1.25	10.05	2.08	1.63
32	Ralph Ellison	23.01	2.09	0.79	22.09	1.68	0.92	24.86	1.13	1.05	11.66	2.20	1.28	13.70	1.67	1.52
33	Michael Ondaatje	22.37	2.09	0.81	17.83	2.84	0.88	19.21	1.61	1.09	10.60	3.93	1.05	10.50	2.62	1.44
34	Raymond Chandler	22.37	1.77	0.89	13.57	1.96	1.34	18.08	1.78	1.09	14.84	3.37	0.87	12.79	1.97	1.46
35	Salman Rushdie	22.37	1.44	1.01	18.60	1.98	1.01	26.55	1.53	0.76	11.66	3.33	1.05	12.33	1.62	1.65
36	Jack London	21.29	2.28	0.82	17.44	1.60	1.23	25.42	1.75	0.75	16.61	3.40	0.78	12.33	1.77	1.57
37	Joyce Carol Oates	20.43	2.50	0.82	16.28	1.84	1.19	14.12	2.02	1.23	15.19	3.47	0.84	12.33	2.79	1.29
38	Kazuo Ishiguro	20.00	1.75	1.01	17.44	1.98	1.08	12.99	1.99	1.31	9.89	4.00	1.09	7.76	1.83	1.96
39	Robert Ludlum	19.14	2.16	0.94	17.44	2.25	1.00	16.38	2.16	1.06	15.90	3.67	0.79	10.96	2.79	1.38
40	Clive Cussler	18.28	1.70	1.11	18.22	1.73	1.12	24.29	1.81	0.78	19.08	2.95	0.72	9.59	3.95	1.35
41	Isabel Allende	17.85	2.22	0.98	16.28	2.60	1.00	23.16	1.55	0.92	9.19	2.70	1.35	9.59	2.13	1.65
42	Willa Cather	16.13	2.72	0.98	12.02	2.01	1.44	14.69	2.38	1.10	8.13	2.93	1.41	11.42	2.02	1.54
43	Thomas Pynchon	15.70	2.46	1.04	16.67	2.15	1.07	21.47	2.02	0.85	12.72	3.51	0.96	11.87	2.22	1.44
44	Jane Smiley	15.05	2.06	1.16	12.40	1.94	1.44	17.51	2.16	1.00	11.31	3.40	1.06	9.13	1.82	1.82
45	Nelson DeMille	15.05	2.49	1.06	16.28	1.68	1.26	16.95	1.36	1.37	15.19	3.38	0.85	12.79	1.73	1.56
46	James Michener	14.84	2.37	1.10	14.73	2.63	1.08	19.77	1.03	1.48	8.83	4.39	1.14	9.13	1.56	1.98
47	Herman Wouk	13.55	1.56	1.46	17.44	1.47	1.30	21.47	1.46	1.04	10.25	3.88	1.08	11.87	2.30	1.42
48	Saul Bellow	13.12	2.75	1.12	13.95	2.74	1.11	15.25	2.44	1.06	9.89	3.20	1.19	7.31	2.60	1.74
49	Bernard Malamud	12.69	2.67	1.15	11.63	2.24	1.40	23.16	2.63	0.69	9.89	2.96	1.24	10.50	1.64	1.80
50	Umberto Eco	12.47	2.90	1.13	18.60	1.97	1.01	12.43	1.83	1.41	8.83	3.34	1.27	12.33	2.60	1.32

The mean number of foils incorrectly identified as authors was low and ranged between 1% and 3% across cohorts

We conducted a correlational analysis that compared the rank-order of accuracy of author recognition across cohorts. A high correlation means that in the two cohorts under comparison, the individual participants tend to more frequently recognize the same authors, while less frequently recognizing others. A low correlation means that readers from different cohorts also vary in the authors they know, and not only the overall levels of familiarity with the authors. Table 6 shows rank-order correlations between percent correct across our five samples. Spearman correlation coefficients are reported above the diagonal, and *p* values below the diagonal. The very high correlation between L1 and L2 English university students in Table 5, alongside the difference in average scores in Table 4, demonstrates that although L2 speakers of English perform lower overall on the ART, there is considerable overlap in the authors they are most familiar with. A similar conclusion can be drawn from the comparisons of L1 and L2 college-level speakers of English. In contrast, the much lower correlation between ESL students and all other cohorts suggests that their responses were more random, which is supported by their comparatively lower average ART score.

**Table 6:** Rank-order correlations between percent correct across five samples. Spearman correlation coefficients are reported above the diagonal, and *p* values below the diagonal

Level of education	English	University			College	
		L1	L2	ESL	L1	L2
University	L1	*****	0.902	0.516	0.867	0.736
	L2	<0.001	*****	0.575	0.810	0.665
	ESL	<0.001	<0.001	*****	0.499	0.446
College	L1	<0.001	<0.001	<0.001	*****	0.771
	L2	<0.001	<0.001	0.001	<0.001	*****

To evaluate the validity of ART for different cohorts, we employed item response theory (IRT), see Moore and Gordon (2015). IRT determines the informative value of each entry in a given test for discriminating the latent ability of participants in some dimension (here, exposure to print, and consequently, reading ability), as well as estimating the overall informativity and the measurement error of the test itself overall (see Embretson & Reise, 2013). To reiterate, our critical question was how valid a test ART is for cohorts widely differing in their exposure to print and reading ability.

A two-parameter IRT model provided a better fit to the data than the one-parameter alternative, the three-parameter alternative, or the Rasch model, as indicated by the likelihood ratio model comparison test. This model returned the difficulty parameter (reflected in the left- vs. rightward shift along the *x*-axis representing latent ability) and the discrimination parameter of the item characteristic curve for every item in the test. The metric of difficulty or the *β* parameter of the model for an individual item (author name) is the estimated level of ability at which an individual would have a greater than 50% probability of correctly responding to this item (recognizing this author). Smaller values of *β* represent lower difficulty. The discrimination *α* parameter is a slope of the line fitted to the item characteristic curve: the steeper it is, the better this item discriminates between responders who give a correct versus incorrect response to this item, see Moore and Gordon (2015) for a detailed description of the two-parameter models.

We fitted the IRT two-parameter models to the ART data of each cohort separately. Table 5 reports the outcomes, comparing all five cohorts by author name for percentage correct, the *α* parameter (discrimination), and the *β* parameter (difficulty), sorted by the percentage each name was correctly selected by university English L1 speakers.

Unsurprisingly, in Table 5 we see that names of authors who are well known to North American audiences top the list of those most likely to be selected by university English L1 speakers (e.g., Steven King, F. Scott Fitzgerald, Ernest Hemingway, or Harper Lee). These are frequently names of authors commonly found in North American public school reading curricula). We also note a relatively high rank of Margaret Atwood in our current Canadian data from L1 university students (rank 3), as compared to that (rank 25) from University of North Carolina (Moore & Gordon, 2015). Margaret Atwood is one of the best known contemporary Canadian fiction writers, and the better recognition of her name in Canada than in the USA aligns with earlier reports of the link between changes in author popularity and ART scores.

In fact, the *β* values show that the native English-speaking university students have an advantage over other cohorts, as these values tend to be lower for them: for example, the 14 most recognized authors show negative values of difficulty (*β*) for the university L1 cohort, while the *β* values for the English L2 college and English ESL students never drop to negative values.

For university English L1 speakers, names with higher *α* parameter values, such as Nelson DeMille, Umberto Eco, Saul Bellow, and Herman Wouk, are more discriminative of latent ability. Put simply, a native English university student who is familiar with these more obscure names can be inferred to have been exposed to more print in their lifetime, and consequently, is more likely to be a more proficient reader.

**Fig. 1:**
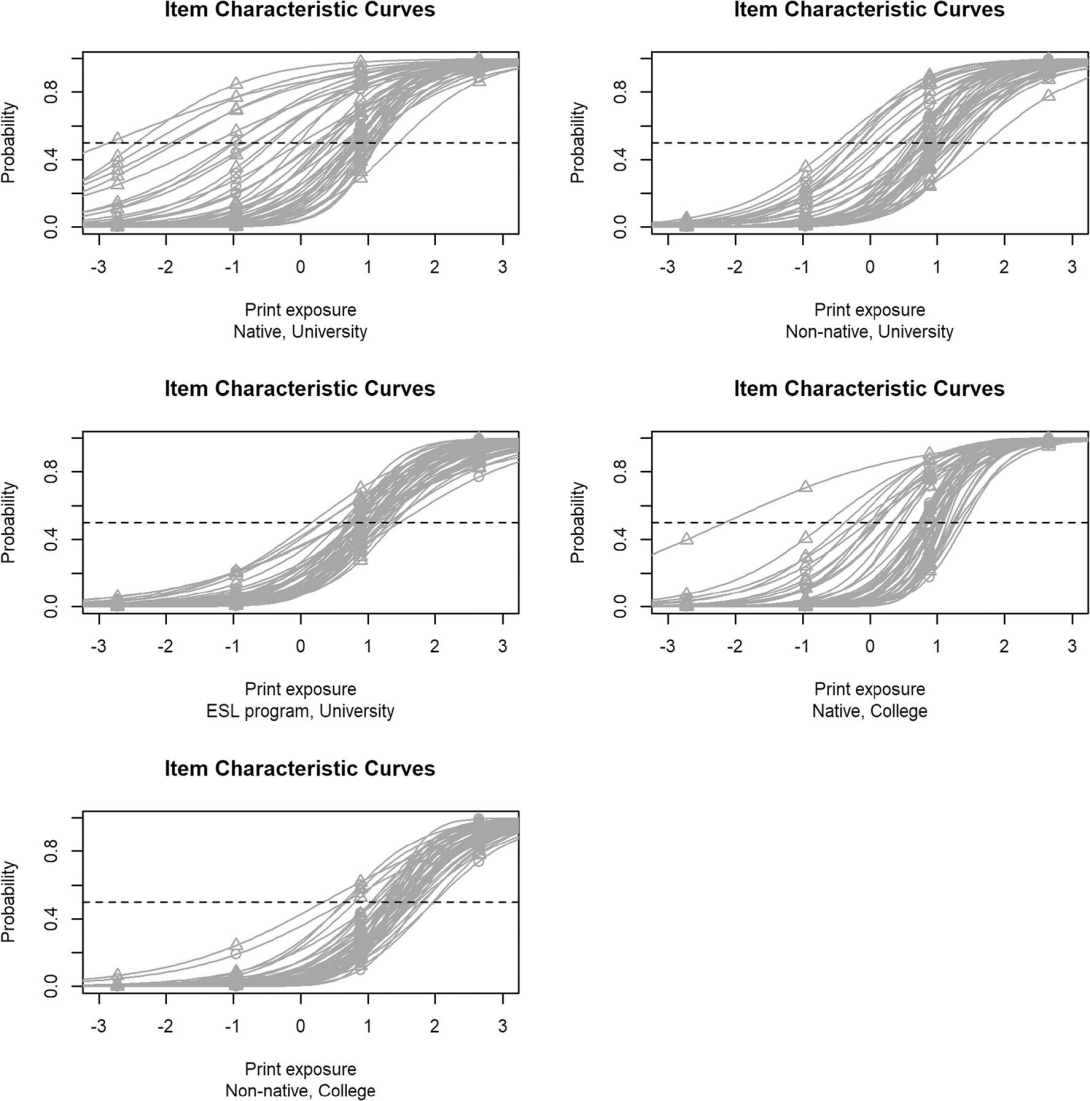
Item characteristic curves comparing all five cohorts

To get a better sense of the unequal distribution of difficulty of the test across cohorts, Fig. 1 visualizes the item estimates of the IRT model for each cohort’s respective item characteristic curves. Each line in each figure represents an individual author’s name from the ART, and the dashed line shows the point at which a participant has a higher or lower than chance probability of correctly selecting the author’s name according to the participant’s latent ability (print exposure). These figures illustrate how a broad range of levels of print exposure can be informative for Native English-speaking university students (top left), whereas in contrast, a very narrow band of name informativity is found for cohorts such as the university ESL program (middle left). This low spread for the ESL readers shows that all items are roughly equally difficult, leading to a floor effect. In general, a rightward shift of curves is observed for most non-native and college students, reflecting the relatively consistent difficulty of most ART items for these cohorts.

**Fig. 2:**
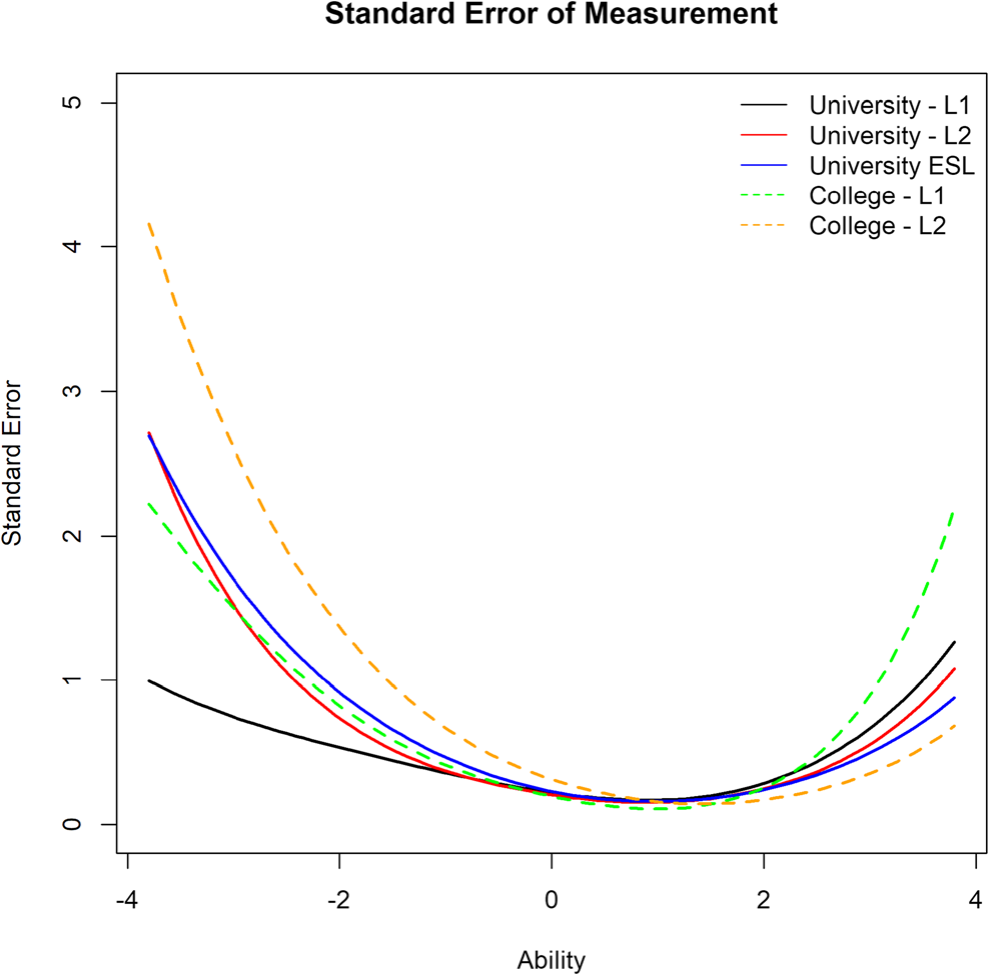
Comparison of standard errors across all five cohorts

An important dimension of a test’s validity is its measurement error. Figure 2 shows a comparison of standard errors across all five cohorts, derived from respective IRT analyses. Item response theory enables us to estimate the standard error of measurement associated with each cohort: Fig. 2 visualizes the estimates as a function of latent ability. These estimates are given on the original scale, i.e., points in the ART score. As expected for a test that appears to demonstrate a floor effect, it is more accurate (has a lower standard error) at the higher levels of latent ability than the lower ones. With the exception of the university L1 cohort, where standard errors were equally low on the two extremes of ability, standard errors were much higher in the lower range of latent ability in all remaining cohorts. For illustration, we report standard errors estimated for the 5th and the 95th percentiles of the distribution of latency (±3.42). For L1 university students, the estimated SE was 0.86 and 0.94, respectively (compare to the mean of 9.95); for L1 college students it was about double in size: 1.85 and 1.45 (with a mean of 7.63 points). Even more drastically, the standard errors for L2 college students were 3.34 and 0.50 points: that is, in the lower range of ability, the standard error exceeded the mean score of 2.92. For ESL participants, the estimates of SE were 2.17 and 0.67 (again, exceeding the mean of 1.45 points in the lower ability range), and for L2 university students the standard errors were 2.06 and 0.79 (with a mean of 5.12 points).

To sum up the findings, for many cohorts, most author names on the ART are highly difficult to recognize, as reflected in the rightward shift of the item characteristic curves in Fig. 1. Additionally, for the L2 cohorts and the ESL cohort, the spread of difficulty is extremely small, suggesting that most ART items are equally difficult, which indicates a floor effect. This is particularly the case for ESL and non-native college cohorts, who seem to be almost equally unfamiliar with all author names on the ART. Also, all cohorts except L1 university students demonstrated a very high standard error of measurement in the test, which makes the mean performance statistically indistinguishable from chance.

In accordance with multiple prior reports, we conclude that the ART is informative and accurate for L1 university students, and we add that it has relatively high informativity and accuracy for L1 college students as well. However, ART is not a reliable or informative tool for L2 students at either the university or college level, and it performs the worst for ESL students.

## General discussion

Exposure to print is one of the most robust predictors of reading performance and reading skill development (see meta-analysis of Mol & Bus, 2011). While primarily discussed in the context of acquisition of literacy in one’s dominant language, it is equally evident that reading more in one’s L2 increases the quality of one’s L2 reading skill as well (Constantino, 1994; Constantino, Lee, Cho, & Krashen, 1997; Gradman & Hanania, 1991; Lao & Krashen, 2000; Mason & Krashen, 1997). Operationalization of exposure to print has a long history of eliciting subjective judgments of the quantity and quality of printed material that a person has access to (reviewed in Stanovich & West, 1989, among others). The introduction of the Author Recognition Test (ART) by Stanovich and West (1989) and derived checklist tests offered a measure that is objective, and one that has been demonstrated among proficient L1 readers as a valid and reliable estimate of one’s reading experience. This paper examined the validity of the ART (developed by Stanovich & West, 1989, and refined by Acheson et al., 2008, and Moore & Gordon, 2015) for readers of English as a first language (L1) and a second language (L2), across levels of English proficiency defined by the educational level (the English-medium university vs. college vs. ESL pre-admission program). The motivation was to verify whether the same test can be meaningfully applied across all these groups as a comparator.

Our findings demonstrate an expected gradient in the ART performance across five cohorts (*N* = 1393), with the decreasing order of performance being as follows: L1 university students, L1 college students, L2 university students, L2 college students, and ESL students. Importantly, all L2 speakers, and especially the groups with lower ART scores, performed virtually at chance level. The IRT analyses confirmed that ART is not informative for any L2 cohort and comes with a relatively high standard error of measurement. The measurement error was also high for L1 college students.

The conclusion is clear: in its current form, ART is not a meaningful test for L2 speakers of English. The factors which contribute to these results are less clear. Specifically, it is unknown whether students with English as L2 are not reading a sufficient amount of material in any language for ART to be an effective measure of print exposure, or whether instead these students are reading different kinds of authors (and in different kinds of languages) than the ones captured by ART. As mentioned in the Introduction, one potential weakness of ART is that the author names selected are all fiction writers generally belonging to the Western school of literature. If some L2 speakers read fiction representing a different tradition or do not read fiction at all, ART may not adequately tap into their “cultural capital” (Bourdieu, 1986; Tunmer & Chapman, 2006). For instance, an adept reader of Chinese literature may not score a single point on ART, because it does not include a single author from that tradition. As far as specific reading in English is concerned, L2 speakers of English enrolled in professional or academic programs may be focusing a greater deal of intellectual energy towards reading textbooks or technical manuals than consuming works of fiction. Again, ART would underestimate their exposure to English print, because it is primarily designed to capture reading for leisure (or “free voluntary reading”) better reflected in reading fiction (Kim & Krashen, 1998b; Lee, Krashen, & Tse, 1997; West, Stanovich, & Mitchell, 1993).

### Limitations

A substantial limitation of the present study is that some of the samples were smaller than samples typically used for estimating IRT parameters (especially university ESL students *N* = 183), while others were adequate in this regard (e.g., L1 university *N* = 466 and college *N* = 283). Our data collection plans were thwarted by the COVID-19 pandemic. While future studies with larger samples may lead to more accurate estimates, we find it unlikely that these estimates will run counter to the present critical finding that ART is an unreliable instrument for L2 readers of English.

### Future directions

In summary, the present results underscore the importance of developing tests of English print exposure which are equally applicable to second language learners as they are to native speakers of English. The Author Recognition Test provides a helpful way of quickly determining exposure to print with a short checklist of items. However, given that the ART varies in validity across cohorts, new methods may be required which put native and non-native speakers of a given language on an equal footing. Preparing an author checklist that would be equally familiar to readers of many languages is not feasible. Adapting ART to a large number of languages is possible, as discussed in the introduction of this paper, but laborious. An additional possibility, which we explore in forthcoming studies, is to task participants with spontaneously naming as many authors as they can within a set period of time. This Author *Naming* Test will require recall and thus would differ from a recognition test like ART, but—with task instructions provided in a person’s L1—it will enable a speaker of any language to tap into their cultural capital in a way unbiased by a specific extraneous literary, linguistic, or cultural tradition. It may also provide a broader representation of a person’s exposure to print by enabling them to identify both fiction authors and names of non-fiction authors, journalists, memoirists, and writers in additional genres. Granted, such a test would face additional challenges, including perhaps some form of external validation of the author names provided. Despite this, if an “author naming test” were to prove to be a reliable determiner of print exposure, it may represent a simpler way of accounting for the many different kinds of reading undertaken by individuals.

#### Open Practices Statement

The data and materials for all experiments are available at https://osf.io/xacbt/?view_only=c2151ae633924b0993ae0bebf7e0a074 and none of the experiments were preregistered.

## Footnotes

1 College is defined in Canada as non-degree-granting postsecondary institution with one- or two-year programs of study.
